# The association between mood state and chronobiological characteristics in bipolar I disorder: a naturalistic, variable cluster analysis-based study

**DOI:** 10.1186/s40345-017-0113-5

**Published:** 2018-02-19

**Authors:** Robert Gonzalez, Trisha Suppes, Jamie Zeitzer, Colleen McClung, Carol Tamminga, Mauricio Tohen, Angelica Forero, Alok Dwivedi, Andres Alvarado

**Affiliations:** 1 Department of Psychiatry, Texas Tech University Health Sciences Center El Paso, El Paso, TX USA; 2 VA Palo Alto Health Care System, Department of Psychiatry and Behavioral Sciences, Stanford University School of Medicine, Palo Alto, CA USA; 3 Department of Psychiatry, University of Pittsburgh Medical Center, Pittsburgh, PA USA; 4 Department of Psychiatry, University of Texas, Southwestern Medical School, Dallas, TX USA; 5 Department of Psychiatry, University of New Mexico Medical School, Albuquerque, NM USA; 6 Department of Biomedical Sciences, Texas Tech University Health Sciences Center El Paso, El Paso, TX USA

**Keywords:** Bipolar disorder, Actigraphy, Rhythm, Sleep, Activity, Cluster analysis, Mood state

## Abstract

**Background:**

Multiple types of chronobiological disturbances have been reported in bipolar disorder, including characteristics associated with general activity levels, sleep, and rhythmicity. Previous studies have focused on examining the individual relationships between affective state and chronobiological characteristics. The aim of this study was to conduct a variable cluster analysis in order to ascertain how mood states are associated with chronobiological traits in bipolar I disorder (BDI). We hypothesized that manic symptomatology would be associated with disturbances of rhythm.

**Results:**

Variable cluster analysis identified five chronobiological clusters in 105 BDI subjects. Cluster 1, comprising subjective sleep quality was associated with both mania and depression. Cluster 2, which comprised variables describing the degree of rhythmicity, was associated with mania. Significant associations between mood state and cluster analysis-identified chronobiological variables were noted. Disturbances of mood were associated with subjectively assessed sleep disturbances as opposed to objectively determined, actigraphy-based sleep variables. No associations with general activity variables were noted. Relationships between gender and medication classes in use and cluster analysis-identified chronobiological characteristics were noted. Exploratory analyses noted that medication class had a larger impact on these relationships than the number of psychiatric medications in use.

**Conclusions:**

In a BDI sample, variable cluster analysis was able to group related chronobiological variables. The results support our primary hypothesis that mood state, particularly mania, is associated with chronobiological disturbances. Further research is required in order to define these relationships and to determine the directionality of the associations between mood state and chronobiological characteristics.

**Electronic supplementary material:**

The online version of this article (10.1186/s40345-017-0113-5) contains supplementary material, which is available to authorized users.

## Background

Chronobiological disturbances are key features of bipolar disorder (Association AP 2013). Empirical data continue to mount implicating these disruptions as components of the pathophysiology of the illness (Gonzalez 2014). A significant body of research suggests a relationship between chronobiological disturbances and mood state in bipolar disorder. For example, disruptions of social rhythms have been associated with the emergence of affective episodes (Malkoff-Schwartz et al. 1998), particularly mania (Malkoff-Schwartz et al. 1998, 2000). A link between psychomotor activity and mood state has also been suggested (Wehr et al. 1980; Teicher 1995). Sleep disturbances have been shown to correlate with affective states (Eidelman et al. 2010) and increased symptom severity (Eidelman et al. 2010; Gruber et al. 2009) and may increase the susceptibility toward developing mania (Bunney et al. 1972; Sitaram et al. 1978). Chronobiological disruptions of physiological functioning have also been noted in relation to affective states in bipolar disorder (Pflug et al. 1981).

Multiple models have been proposed to explain the chronobiological disruptions associated with bipolar disorder (Gonzalez 2014). Some of the most compelling evidence indicates an inherent instability in the biological rhythms of those suffering from the illness. In support of this premise is the wide degree of variability in biological rhythms that has been reported in bipolar disorder (Pflug et al. 1981; Krane-Gartiser et al. 2014; Jones et al. 2005). This variability has been observed in the psychomotor activity patterns related to the illness. For example, patients suffering from bipolar disorder demonstrate greater variability (Krane-Gartiser et al. 2014; Jones et al. 2005), less stable rhythmicity (Krane-Gartiser et al. 2014; Jones et al. 2005), greater fragmentation (Jones et al. 2005), and lower relative activity amplitude (Rock et al. 2014) in locomotor activity when compared to controls. Disturbances in the rhythmic expression of locomotor activity have also been reported in affective states in bipolar disorder (Salvatore et al. 2008; Gonzalez et al. 2014). Prior research has demonstrated that a greater severity of manic symptoms is related to less robust locomotor activity rhythms (Gonzalez et al. 2014). These findings have led some to hypothesize that less robust rhythms are a predisposing factor toward developing mood state symptomatology in patients suffering from affective disorders (Reinberg et al. 2007).

Various chronobiological disturbances have been reported in bipolar disorder, including characteristics associated with general activity levels, sleep, and rhythmicity. There is a paucity of research examining how these variables are interrelated and how they are collectively associated with clinical characteristics of the illness. Variable cluster analysis is a method for grouping variables based on the similarity of their characteristics and identifies the most representative variables in clusters. This statistical approach is a dimension reduction technique, similar in approach to principal component analysis. Variable cluster analysis not only identifies the interrelationships between variables, but also overcomes the problem of interpretation related to principal component analysis. The identification of subgroups via general cluster analysis is relatively common. For example, cluster analysis has been used to identify patient subgroups in affective disorders based on chronobiological characteristics in order to determine relationships with physiological markers of rhythmicity (Carpenter et al. 2017). However, no previous studies have used a variable cluster analysis approach to determine the most salient chronobiological characteristics among a large dimension of variables in association with mood state. While previous studies have focused on the individual relationships between affective state and chronobiological characteristics, to the best of our knowledge, no study has yet to assess how these different types of chronobiological disturbances may be interrelated in their association with mood state. The aim of this study was, therefore, to conduct a variable cluster analysis in order to ascertain how chronobiological traits collectively are associated with affect. Our goal in conducting a variable cluster analysis was also to reduce the number of related variables associated with mood state and to determine the chronobiological markers most representative of these relationships. Based on our previous study that demonstrated a relationship between a lower degree of robustness in the rhythmicity of psychomotor activity and the severity of mania (Gonzalez et al. 2014), we hypothesized that a greater degree of manic symptomatology is associated disturbances of rhythm.

## Methods

### Study description

The protocol is a 1-week naturalistic study designed to assess chronobiological characteristics using actigraphy and clinical measures in bipolar I disorder (BDI) subjects under ambulatory conditions.

### Subjects

DSM-IV-TR Axis I diagnosis of BDI was confirmed by the Structured Clinical Interview for DSM-IV Axis I Disorders (SCID-I/P) (Ventura et al. 1998) or Mini International Neuropsychiatric Interview (MINI) (Sheehan et al. 1998). Subjects with a history of neurological impairment or uncontrolled medical conditions with the potential to disrupt biological rhythms, current use of hypnotic agents for sleep, and a history of substance abuse or dependence 1 month prior to study participation were excluded from the study. Subjects with current lifestyle demands (i.e., work, school) requiring shift work or diurnal changes in work schedule and those undergoing travel involving three or more time zones occurring 4 weeks prior to or during the course of the study were also excluded from the participation. Patients were recruited from county and community hospitals, university medical centers, and community mental health clinics between the dates of March 2009 to January 2016. The study was approved by the University of Texas Southwestern Medical Center, University of Texas Health Sciences Center San Antonio, and Texas Tech University Health Sciences Center El Paso institutional review boards and was consistent with standard for the ethical conduct of human research. Written informed consent was obtained for all subjects. In addition to the previous reported sample (Gonzalez et al. 2014), an additional 63 BDI subjects were included for analysis. Given the increased number of variables assessed and in order to ensure consistency, analyses defining chronobiological characteristics were conducted de novo in all subjects.

### Demographic and clinical characteristics

For purposes of cofactor analysis, information regarding age, gender, and current medication status, including the classes of medications currently in use, was collected. The severity of manic and depressive symptoms was defined by using the Young Mania Rating Scale (Young et al. 1978) and 30-Item Inventory of Depressive Symptomatology (Rush et al. 1996), respectively.

### Clinical assessments of sleep and rhythm

Clinical assessments were also used to assess sleep and rhythm characteristics. These included the 5-Item Social Rhythm Metric (SRM-5) (Monk et al. 2002), designed to capture the degree of lifestyle regularity, and the Pittsburgh Sleep Quality Index (PSQI) (Buysse et al. 1989) that characterizes general sleep health (PSQI scores > 5 are associated with disrupted sleep). Sleep diaries were collected to help confirm estimated sleep periods (Gonzalez et al. 2013).

### Actigraphy

Basic Motionlogger actigraph units (Ambulatory Monitoring, Inc., Ardsley, NY) were used to collect data regarding locomotor activity. Subjects wore actigraph units on the non-dominant wrist. Data were recorded in proportional integral mode (PIM) and sampled in 60-s epochs. Summary statistics characterizing psychomotor activity, sleep parameters, and rhythmicity were calculated from the raw motor activity data using Action 4 circadian rhythm analysis software and Action W-2 software (Ambulatory Monitoring, Inc., Ardsley, NY).

Summary statistics assessing the intensity and rate of change of physical activity included *activity mean* (average daily minutes of physical activity), *activity median* (median daily minutes of physical activity), *acceleration index* (change in activity rate during interval; an estimate of physical activity intensity), and *activity index* (percentage of time in minutes where activity levels were recorded at greater than zero). Sleep-based statistics included *sleep percent* (average of the percentage of minutes scored as sleep), and *sleep efficiency* (proportion of the total sleep time to time in bed), and *average daily sleep time* (sleep time; average daily minutes scored as sleep). Rhythm-based variables included *amplitude* (difference between the peak and the mean activity values as determined via cosinor analysis), *mesor* (circadian rhythm-adjusted mean activity value based on cosinor analysis), *circadian quotient* (CQ; cosinor analysis-based amplitude to mesor ratio; an estimation of how well circumscribed periods of activity are during the course of the day and used as a proxy for the robustness of rhythms), *goodness*-*of*-*fit* (GOF; measure of how well the activity data fits the cosine function which can be taken as an indicator of strength of rhythm), and *24*-*h correlation* (autocorrelation of a time series with its own past and future values that may be taken as an indicator of the degree of rhythmicity), *interdaily stability* (IS; the stability of activity rhythm between days), *intradaily variability* (IV; fragmentation of activity within a 24-h period), and *relative amplitude* (RA; average for the ratio of the most active 10-h period and least active 5-h period).

### Statistical analysis

Descriptive statistics were generated for demographic and sample characteristics, actigraphic assessments, and clinical scales. Quantitative variables were described using mean and standard deviation (SD). Categorical variables were summarized using frequencies and percentages.

In order to reduce the dimension and determine clustering of chronobiological variables, a variable cluster analysis (proc varclus) was conducted. Cluster scores were generated using the standardized scores for the chronobiological variables and the coefficients obtained from the principal component method. The representative variable for each cluster (smallest 1*−R*^2^ within each cluster) was also identified. Ordinary linear regression analyses adjusting for age, gender, and medication classes in use (lithium, anticonvulsants, antidepressants, antipsychotics, and benzodiazepines) were conducted to determine the associations between mood state, as defined by YMRS and IDS-30-C scores, and identified clusters. To assess the relationships between mood state and variable cluster analysis-identified chronobiological characteristics, both Pearson’s correlations and adjusted linear regression analyses were conducted. In order to determine the effects of multiple testing, these analyses were also conducted with all 17 chronobiological variables considered individually. Benjamini–Hochberg procedure was used to adjust for false-positive probability in the adjusted linear regression modeling. All the statistical analyses were carried out using SAS 9.4. *p* values less than or equal to 5% were considered as significant variables.

## Results

### Sample characteristics

A total of 105 BDI subjects were included for analysis. Sample characteristics are summarized in Table 1. The cohort was mostly middle-aged, Caucasian, and female, with most participants being on psychiatric medications during the study. The average YMRS and IDS-30-C scores were 17.44 ± 9.16 and 23.80 ± 11.61, respectively. This reflects mania in the mild-to-moderate range and depression of moderate severity. The mean PSQI and SRM-5 scores were 10.30 ± 4.04 and 3.79 ± 1.30, respectively. The majority of the sample (91.35%) had a PSQI score > 5 which is indicative of disrupted sleep. The mean observational period was 7.5 days. Subjects were taking a mean of 1.95 ± 1.13 psychiatric medications (median = 2, minimum = 0, maximum = 4).

**Table 1:** Sample characteristics

	Mean ± standard deviation or *N* (%)
Demographic characteristics	
Age (in years)	41.20 ± 11.34
Gender	
Female	66 (62.86%)
Male	39 (37.14%)
Race	
Caucasian	57 (54.29%)
Hispanic	35 (33.33%)
African-American	13 (12.38%)
Clinical characteristics	
Mood state	
YMRS	14.62 ± 9.27
IDS-30-C	24.52 ± 12.18
Psychiatric medication status	
Medicated	90 (86.54%)
Unmedicated	14 (13.64%)
Lithium	19 (18.10%)
Anticonvulsants	56 (53.33%)
Antidepressants	45 (42.86%)
Antipsychotics	55 (52.38%)
Benzodiazepines	26 (24.76%)
Actigraphic assessments	
Interdaily stability (IS)	0.52 ± 0.15
Intradaily variability (IV)	0.67 ± 0.20
Relative amplitude (RA)	0.80 ± 0.14
Goodness-of-fit (GOF)	0.46 ± 0.13
Sleep efficiency	66.02 ± 13.55
Sleep percent	31.42 ± 7.08
Average daily sleep time (in hours)	7.49 ± 2.19
24-h correlation	0.25 ± 0.13
Mesor	2925.24 ± 1604.64
Amplitude	2159.33 ± 1235.05
Circadian quotient (CQ)	0.74 ± 0.17
Activity mean	2843.84 ± 1565.51
Activity median	1852.60 ± 1614.06
Acceleration index	38.26 ± 115.35
Activity index	65.48 ± 29.87
Clinical chronobiological assessments	
5-Item Social Rhythm Metric (SRM-5)	3.79 ± 1.30
Pittsburgh Sleep Quality Index (PSQI)	10.30 ± 4.04

Summarizes the sample characteristics. This includes demographic information, clinical characteristics, and actigraphic measurements

*YMRS* Young Mania Rating Scale, *IDS-30-C* 30-Item Inventory of Depressive Symptomatology

### Clustering of chronobiological markers

Variable cluster analysis identified five cluster groups (Table 2). Cluster groups were characterized by subjective sleep disruption (Cluster 1), the rhythmicity and regularity of daily activity patterns (Cluster 2), rate of change and magnitude of movement (Cluster 3), objective measurements of sleep (Cluster 4), and the fitted daily activity amplitude, activity levels and fragmentation of activity within a 24-h period (Cluster 5). The most representative variables for Clusters 1 through 5 were PSQI, IS, acceleration index, sleep time, and mesor, respectively. The variation and proportion explained by the five clusters was 12.77 and 75.0%, respectively.

**Table 2:** Cluster analysis results

Cluster	Variable	Standardized scoring coefficients	*R*-squared with		1−*R*^2^	Variation explained by clusters	Proportion explained by clusters
			Own	Next	Ratio		
			Cluster	Closest			
Cluster 1	PSQI	1.00	1.00	0.08	0.00*	6.12	0.36
Cluster 2	IS	0.23	0.84	0.25	0.22*	8.6	0.51
	RA	0.21	0.69	0.16	0.37		
	GOF	0.24	0.86	0.48	0.28		
	24-h correlation	0.22	0.74	0.40	0.43		
	CQ	0.20	0.61	0.21	0.49		
	SRM-5	0.11	0.18	0.05	0.87		
Cluster 3	Acceleration index	0.53	0.90	0.01	0.10*	10.36	0.61
	Activity index	− 0.53	0.90	0.04	0.11		
Cluster 4	Sleep efficiency	0.31	0.38	0.07	0.66	11.87	0.70
	Sleep percent	0.45	0.82	0.21	0.23		
	Sleep time	0.45	0.80	0.05	0.21*		
Cluster 5	IV	0.24	0.44	0.28	0.77	12.77	0.75
	Mesor	0.23	0.95	0.15	0.05*		
	Amplitude	0.23	0.88	0.39	0.20		
	Activity mean	0.23	0.91	0.13	0.11		
	Activity median	− 0.16	0.88	0.16	0.14		

The results of the principal components-based cluster analysis (proc varclus) of chronobiological variables. Five cluster groups were identified. Summarized are the variables that comprise the clusters, the standardized scoring coefficients, and the representative chronobiological variables for each cluster (smallest 1−*R*^2^ within each cluster). * and italic font denotes most representative variable in the cluster. Presented are the values for the variation and proportion of the variation explained in the sample based on the number of chronobiological clusters considered. Demonstrated are the variation and proportion of variation explained in the sample when considering successive clusters. The total variation and total proportion of the sample explained when including all five clusters were 12.77 and 75.00%, respectively

*IS* interdaily stability, *IV* intradaily variability, *RA* relative amplitude, *CQ* circadian quotient, *GOF* goodness-of-fit, *SRM-5* 5-Item Social Rhythm Metric, *PSQI* Pittsburgh Sleep Quality Index

### Relationships between mood state and identified clusters

After adjusting for age, gender, and medication class, Cluster 1, comprising solely PSQI, was positively associated with both YMRS (RC = 0.027, *p* = 0.012) and IDS-30-C (RC = 0.024, *p* = 0.005) scores. Cluster 2 was negatively associated with YMRS scores (RC = − 0.033, *p* = 0.0001). In addition, female gender was associated with increased Cluster 2 score (RC = 0.461, *p* = 0.016).

### Associations between mood state and individual chronobiological characteristics

The results from analyses assessing the relationship between mood state and cluster analysis-identified chronobiological variables are represented in Table 3. On unadjusted Pearson’s correlations, YMRS was negatively correlated with all variable cluster analysis-identified variables and IDS-30-C was associated with PSQI. After adjusting for age, gender, and medication class, YMRS was negatively associated with IS, RA, GOF, CQ, and PSQI with a trend toward association with SRM-5. IDS-30-C was positively associated with PSQI. After correcting for possible false discovery rate, YMRS remained significantly associated with IS, RA, CQ with a trend toward significance with PSQI. IDS-30-C remained associated with PSQI. In this modeling, associations between variable cluster analysis-identified chronobiological characteristics and both gender and medication class were noted (Table 4). A positive association was noted with female gender. Lithium was positively associated with several markers of rhythm. Antidepressants demonstrated negative associations with rhythm markers and positive associations with PSQI scores. Both variable cluster analysis and individual variable analyses identified the same associations between mood state and chronobiological characteristics (Additional file 1: Table S1). Given the reduction in the number of variables, the variable cluster analysis approach proved to be more powerful when correcting for false discovery rate. Since subjects were on a variable number of medications, exploratory analyses were conducted to assess the impact that the number of medications had on the expression of chronobiological variables. These analyses demonstrated that the class of psychiatric medication used had a greater effect on the associations between mood state and chronobiological characteristics than did the number of medications currently in use (Additional file 2: Table S2).

**Table 3:** Relationships between mood and cluster analysis-identified chronobiological characteristics

Variables	YMRS			IDS-30-C		
	Unadjusted correlations *r* (*p* value)	Adjusted linear regression RC (*p* value)	BHC *p* value	Unadjusted correlations *r* (*p* value)	Adjusted linear regression RC (*p* value)	BHC *p* value
IS	− *0.331* (*0.0007*)*	− *0.004* (*0.005*)*	0.030*	0.0005 (0.995)	0.001 (0.25)	0.956
RA	− *0.391* (< *0.0001*)*	− *0.005* (*0.0003*)*	0.0021*	− 0.064 (0.523)	0.001 (0.268)	0.956
GOF	− *0.333* (*0.0006*)*	− *0.003* (*0.017*)*	0.051^a^	− 0.099 (0.32)	− 0.00 (0.956)	0.956
CQ	− *0.217* (*0.027*)*	− *0.005* (*0.006*)*	0.030*	0.057 (0.565)	0.002 (0.181)	0.956
24-h correlation	− *0.317* (*0.001*)*	− 0.003 (0.623)	0.623	− 0.152 (0.124)	− 0.00 (0.623)	0.956
SRM-5	− *0.218* (*0.0325*)*	− 0.026 (0.09)^a^	0.180	− 0.038 (0.696)	0.004 (0.741)	0.956
PSQI	*0.351* (*0.0003*)*	*0.111* (*0.012*)*	0.048*	*0.396* (< *0.0001*)*	*0.099* (*0.005*)*	0.035*

The relationships between mood rating scale scores and variables characterizing biorhythms. Unadjusted correlations as well as linear regression results adjusted for age, gender, and medication class use (lithium, anticonvulsants, antidepressants, antipsychotics, benzodiazepines) are shown. *r* denotes correlation coefficient and RC denotes regression coefficient

*IS* interdaily stability, *IV* intradaily variability, *RA* relative amplitude, *CQ* circadian quotient, *GOF* goodness-of-fit, *SRM-5* 5-Item Social Rhythm Metric, *PSQI* Pittsburgh Sleep Quality Index, *YMRS* Young Mania Rating Scale, *IDS-30-C* 30-Item Inventory of Depressive Symptomatology. *BHC* Benjamini–Hochberg Correction for multiple testing

* and italic font denotes statistical significance

^a^Denotes a trend toward significance

**Table 4:** Associations between cofactors and chronobiological variables

	IS RC (*p* value)	RA RC (*p* value)	GOF RC (*p* value)	CQ RC (*p* value)	24-h correlation RC (*p* value)	SRM-5 RC (*p* value)	PSQI RC (*p* value)
Medications							
Li	0.07 (0.054)^a^	0.06 (0.082)^a^	*0.07* (*0.037*)*	0.04 (0.377)	*0.07* (*0.023*)*	0.11 (0.725)	1.57 (0.127)
AC	− 0.03 (0.20)	− 0.03 (0.255)	− 0.02 (0.335)	0.008 (0.806)	− 0.03 (0.228)	− 0.06 (0.813)	− 0.18 (0.821)
AD	− 0.05 (0.078)^a^	− *0.07* (*0.004*)*	− *0.05* (*0.031*)*	− 0.04 (0.178)	− *0.05* (*0.03*)*	− 0.39 (0.128)	1.46 (0.07)^a^
AP	0.00 (0.974)	0.02 (0.322)	− 0.02 (0.355)	− 0.03 (0.322)	− 0.03 (0.168)	0.05 (0.859)	− 0.37 (0.646)
BZD	− 0.03 (0.378)	− 0.03 (0.295)	− 0.01 (0.691)	− 0.01 (0.766)	− 0.01 (0.751)	− 0.42 (0.15)	1.24 (0.176)
Other covariates							
Age	0.0004 (0.75)	− 0.001 (0.198)	− 0.0003 (0.769)	− 0.0002 (0.86)	− 0.001 (0.272)	0.014 (0.19)	0.042 (0.235)
Gender (female)	*0.074* (*0.015*)*	0.052 (0.062)^a^	*0.072* (*0.004*)*	*0.09* (*0.007*)*	*0.054* (*0.034*)*	0.172 (0.517)	0.23 (0.779)

The table highlights the associations between cofactors and chronobiological variables identified via cluster analysis. Results are presented as regression coefficients (RC) and associated *p* values

*Li* lithium, *AC* anticonvulsants, *AD* antidepressants, *AP* antipsychotics, *BZD* benzodiazepines. *IS* interdaily stability, *IV* intradaily variability, *RA* relative amplitude, *CQ* circadian quotient, *GOF* goodness-of-fit, *SRM-5* 5-Item Social Rhythm Metric, *PSQI* Pittsburgh Sleep Quality Index

* and italic font denotes statistical significance

^a^Denotes a trend toward significance

## Discussion

To the best of our knowledge, this is the first study to examine the application of variable cluster analysis in determining the relationships between mood state and chronobiological characteristics in BDI. Variable cluster analysis was able to successfully identify and cluster chronobiological variables with related characteristics. A strength of this approach was highlighted by the ability of the variable cluster analysis to group variables characterizing similar phenomena even with variables being calculated via different mathematical models and algorithms (Ancoli-Israel et al. 2003). By implementing a variable cluster analysis, the number of targeted variables was able to be significantly reduced. This reduction in the number of variables increased the power to detect significant associations between mood state and chronobiological variables secondary to the more favorable multiple testing adjustment profile. Furthermore, this technique was able to identify the most representative variables associated with their respective clusters.

The findings of this study support our overarching hypothesis that mania is associated with disturbances of rhythm. Cluster 2, which comprised variables describing the robustness and degree of rhythmicity, was associated with mania. While all six variables in Cluster 2 demonstrated associations with mania, two of these variables may be of particular importance. IS was significantly associated with YMRS scores in all analyses and was identified by cluster analysis as the most representative variable in its cluster. Lower IS has been associated with bipolar disorder subjects as compared to controls (Jones et al. 2005) with evidence suggesting that this actigraphy variable may have predictive value for determining the risk of relapse (Novak et al. 2014). A recent study also identified IS as a potential phenotype in bipolar disorder pedigrees with an associated locus on chromosome 12 (Pagani et al. 2016). RA was also significantly associated with YMRS scores on all analyses. This variable demonstrated the highest degree of significance in association with mania. A lower RA has been reported in bipolar disorder subjects as compared to controls (Rock et al. 2014; Ng et al. 2015) and in individuals at risk for developing the illness (Ankers and Jones 2009).

Study findings also showed associations between mood state and subjective assessments of sleep. Cluster 1, comprising subjective sleep quality as measured via PSQI, was associated with both mania and depression. While subjectively assessed sleep disturbances were associated with the severity of affective symptoms, no associations between objectively determined, actigraphy-based sleep variables and affective states were noted. Although contrary to some previous reports (Eidelman et al. 2010a, b; Gruber et al. 2009; Hudson et al. 1992), our findings are in line with research demonstrating a discrepancy between objective and subjective assessments of sleep in patients suffering from bipolar disorder (Gonzalez et al. 2013; Harvey et al. 2005; Armitage et al. 1997; Rotenberg et al. 2000; Tsuchiyama et al. 2003) and suggest that rhythm disturbances may be a more robust characteristic of the disorder than sleep disturbances (Jones et al. 2005).

In our sample, we found no relationships between mood state and variables characterizing intensity or rate of change of activity. While not in accord with some previous studies (Wehr et al. 1980; Teicher 1995; Klein et al. 1992), the findings suggest, as other investigators have reported, that it is a less robust rhythm of psychomotor activity that is a key characteristic of the illness (Krane-Gartiser et al. 2014; Jones et al. 2005; Rock et al. 2014) and one which is associated with mania (Gonzalez et al. 2014).

The results also point to additional factors that may impact the expression of biological rhythms in bipolar disorder. For example, relationships between gender and chronobiological characteristics were demonstrated. Given other studies which suggest that gender and chronobiological characteristics can mediate the expression of mood disorders (White et al. 2016), further research is required to determine these relationships. Psychiatric medications can significantly alter chronobiology in this clinical population (Dallaspezia and Benedetti 2015). Our findings indicate that medication class, more than the number of medications in use, accounted for these effects. Results from statistical modeling testing the relationships between mood state and cluster analysis-identified chronobiological variables noted that lithium may have a stabilizing effect on rhythms while antidepressants may have the opposite effect (Dallaspezia and Benedetti 2015). Lithium has been found to lengthen the circadian periods (Johnsson et al. 1983). These relationships may have clinical and therapeutic implications. For example, in patients suffering from bipolar depression, lithium has been associated with phase delays (Campbell et al. 1989). In addition, bipolar patients with a shorter circadian period may respond positively to lithium (Kripke et al. 1978). Lithium may also exert its therapeutic effects by decreasing the sensitivity of melatonin secretion to nocturnal light exposure (Hallam et al. 2005). Some antidepressants have been shown to cause phase advancements (Sprouse et al. 2006) and shorten circadian periods (Nomura et al. 2008). Antidepressants, however, can have a variable impact on chronobiology (Duncan 1996). Interestingly, antidepressants that cause phase delays have been associated with improvements in sleep continuity (Duncan 1996).

## Limitations and future directions

While promising, these results must be viewed in the context of the studies limitations and with an eye on future areas of research. A primary study limitation is the cross-sectional design that does not allow us to infer causal relationships between rhythm disturbances and mood state. Longitudinal studies are, therefore, required to determine the directionality and the temporal relationship between mood and chronobiological characteristics in bipolar disorder. Some limitations are associated with the naturalistic design of the study. Subsequent studies should more carefully account for clinical and demographic characteristics, such as social schedules (i.e., school and or employment, family responsibilities, weekend or holiday schedules) and clinical factors (i.e., body mass index), which may impact chronobiology in the illness. The use of psychiatric medications is also a limitation of the study. While we were able to account for the medication class and number of psychiatric medications used, we were not able to examine dosages or the complexity of medication regimens. Since various psychiatric medications can alter chronobiological functioning (Duncan 1996), future research should aim to address this limitation. Also, it should be noted that rhythmicity of locomotor activity is only considered a proxy for circadian rhythmicity. More definitive studies (Hida et al. 2012) are, therefore, required to fully characterize physiological markers of circadian rhythms.

## Conclusions

Variable cluster analysis proved to be a statistical methodology able to group chronobiological variables based on related characteristics in BDI. These clusters and the individual markers comprising them were able to demonstrate relationships with affective symptomatology. The findings of the present study are in line with a growing body of literature identifying a relationship between chronobiological disturbances and bipolar disorder. The results support our primary hypothesis that mania is associated with rhythm disturbances. Both depression and mania were also associated with subjective assessments of sleep. The findings also suggest that other demographic and clinical characteristics, such as gender and psychiatric medication use, may affect the relationship between chronobiology and mood state. Further research is required in order to define these relationships and to determine the directionality of the associations between mood state and chronobiological disruptions in bipolar disorder.

## Additional files

**Additional file 1: Table S1.** Relationships between mood and all chronobiological characteristics.

**Additional file 2: Table S2.** Relationships between mood and cluster analysis-identified chronobiological characteristics with number of psychiatric medications considered.

## Abbreviations

BDI: bipolar I disorder
IS: interdaily stability
IV: intradaily variability
RA: relative amplitude
CQ: circadian quotient
GOF: goodness-of-fit
SRM-5: 5-Item Social Rhythm Metric
PSQI: Pittsburgh Sleep Quality Index
YMRS: Young Mania Rating Scale
IDS-30-C: 30-Item Inventory of Depressive Symptomatology
*r*: correlation coefficient
RC: regression coefficient
