# Supplementary material for: The association between mood state and chronobiological characteristics in bipolar I disorder: a naturalistic, variable cluster analysis-based study
Source: Int J Bipolar Disord. 2018 Feb 19;6:5. doi: 10.1186/s40345-017-0113-5 (PMC6161964; doi:10.1186/s40345-017-0113-5)
Supplement: Supplementary file 1 — Additional file 1: Table S1. Relationships between mood and all chronobiological characteristics. [file 40345_2017_113_MOESM1_ESM.docx]

|  | **YMRS** | |  | **IDS-30-C** | |  |
| --- | --- | --- | --- | --- | --- | --- |
| **Variables** | **Unadjusted**  **Correlations**  **r (p-value)** | **Adjusted**  **Linear Regression RC (p-value)** | **BHC**  **P-value** | **Unadjusted**  **Correlations**  **r (p-value)** | **Adjusted**  **Linear Regression RC (p-value)** | **BHC**  **P-value** |
| **IS** | **-0.331 (0.0007)*** | **-0.004 (0.005)*** | 0.08**^✜^** | 0.0005 (0.995) | 0.001 (0.25) | 0.956 |
| **IV** | -0.014 (0.886) | -0.002 (0.249) | 0.975 | 0.047 (0.638) | 0.0007 (0.699) | 0.956 |
| **RA** | **-0.391 (<.0001)*** | **-0.005 (0.0003)*** | **0.005*** | -0.064 (0.523) | 0.001 (0.268) | 0.956 |
| **Amplitude** | -0.173 (0.079) | -16.54 (0.227) | 0.975 | -0.162 (0.1) | -13.74 (0.213) | 0.956 |
| **Mesor** | -0.097 (0.325) | -0.721 (0.968) | 0.975 | -0.186 (0.059)**^✜^** | -27.43 (0.061)**^✜^** | 0.956 |
| **GOF** | **-0.333 (0.0006)*** | **-0.003 (0.017)*** | 0.221 | -0.099 (0.32) | -0.00 (0.956) | 0.956 |
| **CQ** | **-0.217 (0.027)*** | **-0.005 (0.006)*** | 0.09**^✜^** | 0.057 (0.565) | 0.002 (0.181) | 0.956 |
| **24-Hour Correlation** | **-0.317 (0.001)*** | -0.003 (0.623) | 0.975 | -0.152 (0.124) | -0.00 (0.623) | 0.956 |
| **SRM-5** | **-0.218 (0.0325)*** | -0.026 (0.09)**^✜^** | 0.975 | -0.038 (0.696) | 0.004 (0.741) | 0.956 |
| **Sleep Efficiency** | -0.005 (0.954) | -0.005 (0.975) | 0.975 | -0.012 (0.903) | 0.069 (0.589) | 0.956 |
| **Sleep Percent** | -0.121 (0.231) | -0.145 (0.074)**^✜^** | 0.888 | 0.049 (0.625) | 0.056 (0.384) | 0.956 |
| **Sleep Time** | -0.032 (0.747) | -1.53 (0.902) | 0.975 | 0.008 (0.936) | -1.74 (0.861) | 0.956 |
| **PSQI** | **0.351 (0.0003)*** | **0.111 (0.012)*** | 0.168 | **0.396 (<0.0001)*** | **0.099 (0.005)*** | 0.085**^✜^** |
| **Activity Mean** | -0.068 (0.494) | 6.1 (0.731) | 0.975 | -0.125 (0.208) | -21.53 (0.135) | 0.956 |
| **Activity Median** | -0.066 (0.504) | 15.06 (0.411) | 0.975 | -0.143 (0.147) | -24.64 (0.097)**^✜^** | 0.956 |
| **Acceleration Index** | -0.033 (0.736) | -0.421 (0.736) | 0.975 | -0.144 (0.144) | -1.64 (0.105) | 0.956 |
| **Activity Index** | 0.132 (0.181) | 0.552 (0.115) | 0.975 | 0.124 (0.209) | 0.084 (0.764) | 0.956 |

**Supplemental Table 1. Relationships between mood and all chronobiological characteristics.** This table summarizes the relationships between mood rating scale scores and all variables characterizing biorhythms included in the study. Unadjusted correlations as well as linear regression results adjusted for age, gender, and medication class use (lithium, anticonvulsants, antidepressants, antipsychotics, benzodiazepines) are shown. ***** and bolded font denotes statistical significance. **^✜^** denotes a trend toward significance. Interdaily Stability (IS), Intradaily Variability (IV), Relative Amplitude (RA), Circadian Quotient (CQ), Goodness-of-Fit (GOF), 5-Item Social Rhythm Metric (SRM-5), Pittsburgh Sleep Quality Index (PSQI), Young Mania Rating Scale (YMRS), 30-Item Inventory of Depressive Symptomatology (IDS-30-C). r denotes correlation coefficient and RC denotes regression coefficient. BHC (Benjamini-Hochberg Correction for multiple testing).
