# Supplementary material for: The association between mood state and chronobiological characteristics in bipolar I disorder: a naturalistic, variable cluster analysis-based study
Source: Int J Bipolar Disord. 2018 Feb 19;6:5. doi: 10.1186/s40345-017-0113-5 (PMC6161964; doi:10.1186/s40345-017-0113-5)
Supplement: Supplementary file 2 — Additional file 2: Table S2. Relationships between mood and cluster analysis-identified chronobiological characteristics with number of psychiatric medications considered. [file 40345_2017_113_MOESM2_ESM.docx]

|  | **YMRS** | |  | **IDS-30-C** | |  |
| --- | --- | --- | --- | --- | --- | --- |
| **Variables** | **Unadjusted**  **Correlations**  **r (p-value)** | **Adjusted**  **Linear Regression RC (p-value)** | **BHC**  **p-value** | **Unadjusted**  **Correlations**  **r (p-value)** | **Adjusted**  **Linear Regression RC (p-value)** | **BHC**  **p-value** |
| **IS** | **-0.331 (0.0007)*** | **-0.005 (0.002)*** | **0.012*** | 0.0005 (0.995) | 0.0009 (0.446) | 0.940 |
| **RA** | **-0.391 (<.0001)*** | **-0.005 (0.0001)*** | **0.007*** | -0.064 (0.523) | 0.0007 (0.504) | 0.940 |
| **GOF** | **-0.333 (0.0006)*** | **-0.003 (0.007)*** | **0.021*** | -0.099 (0.32) | -0.0004 (0.705) | 0.940 |
| **CQ** | **-0.217 (0.027)*** | **-0.005 (0.003)*** | **0.015*** | 0.057 (0.565) | 0.001 (0.244) | 0.940 |
| **24-Hour Correlation** | **-0.317 (0.001)*** | **-0.003 (0.014)*** | **0.028*** | -0.152 (0.124) | -0.0008 (0.433) | 0.940 |
| **SRM-5** | **-0.218 (0.0325)*** | **-0.029 (0.047)*** | **0.047*** | -0.038 (0.696) | 0.0008 (0.94) | 0.940 |
| **PSQI** | **0.351 (0.0003)*** | **0.119 (0.005)*** | **0.020*** | **0.396 (<0.0001)*** | **0.108 (0.001)*** | **0.007*** |

**Supplemental Table 2. Relationships between mood and cluster analysis-identified chronobiological characteristics with number of psychiatric medications considered.** Supplemental Table 2 summarizes the correlations between mood rating scale scores and variables characterizing biorhythms. Unadjusted correlations as well as linear regression results adjusted for age, gender, and the number of medications are shown. ***** denotes statistical significance. Interdaily Stability (IS), Intradaily Variability (IV), Relative Amplitude (RA), Circadian Quotient (CQ), Goodness-of-Fit (GOF), 5-Item Social Rhythm Metric (SRM-5), Pittsburgh Sleep Quality Index (PSQI), Young Mania Rating Scale (YMRS), 30-Item Inventory of Depressive Symptomatology (IDS-30-C). r denotes correlation coefficient and RC denotes regression coefficient.
